# MDS shows a higher expression of hTERT and alternative splice variants in unactivated T-cells

**DOI:** 10.18632/oncotarget.12115

**Published:** 2016-09-19

**Authors:** Wen Dong, Lei Wu, Houfang Sun, Xiubao Ren, Pearlie K. Epling-Burnette, Lili Yang

**Affiliations:** ^1^ Department of Orthopaedic Surgery, Tianjin Hongqiao Hospital, Tianjin, P.R. China; ^2^ Department of Immunology, Tianjin Cancer Institute and Hospital, Tianjin Medical University, P.R. China; ^3^ National Clinical Research Center of Cancer, P.R. China; ^4^ Key Laboratory of Cancer Immunology and Biotherapy, Tianjin, P.R. China; ^5^ Immunology Program at the H. Lee Moffitt Cancer Center, Tampa, FL, USA

**Keywords:** MDS, T-cells, telomerase, hTERT, hTERT alternative splice variants

## Abstract

Telomere instability and telomerase reactivation are believed to play an important role in the development of myelodysplastic syndromes (MDS). Abnormal enzymatic activity of human telomerase reverse transcriptase (hTERT), and its alternative splice variants have been reported to account for deregulated telomerase function in many cancers. In this study, we aim to compare the differences in expression of hTERT and hTERT splice variants, as well as telomere length and telomerase activity in unstimulated T-cells between MDS subgroups and healthy controls. Telomere length in MDS cases was significantly shorter than controls (n = 20, *p*<0.001) and observed across all subtypes of MDS using World Health Organization classification (WHO subgroups versus control: RARS, *p*= 0.009; RCMD, *p*=0.0002; RAEB1/2, *p*=0.004, respectively) and the International Prognostic Scoring System (IPSS subgroups: Low+Int-1, *p*<0.001; Int-2+High, *p*=0.004). However, unstimulated T-cells from MDS patients (n=20) had significantly higher telomerase activity (*p*=0.002), higher total hTERT mRNA levels (*p*=0.001) and hTERT α+β- splice variant expression (*p*<0.001) compared to controls. Other hTERT splice variants were lower in expression and not significantly different among cases and controls. Telomerase activity was positively correlated with total hTERT levels in MDS (r=0.58, *p*=0.007). This data is in sharp contrast to data published previously by our group showing a reduction in telomerase and hTERT mRNA in MDS T-cells after activation. In conclusion, this study provides additional insight into hTERT transcript patterns and activity in peripheral T-cells of MDS patients. Additional studies are necessary to better understand the role of this pathway in MDS development and progression.

## INTRODUCTION

MDS represents a diverse group of clonal disorders characterized by impaired proliferation and differentiation of hematopoietic stem cells or progenitor cells, dysplastic morphology, ineffective haematopoiesis, and an elevated risk of progression into acute myeloid leukemia (AML)[1]. Many decades of research have documented robust association between abnormal telomere dynamics and clinical outcome in MDS patients, whereby it has been well established that telomere shortening represented one of the most highly representative features of MDS predicting for poor prognosis [2, 3].

Telomeres are a repetitive hexanucleotide (TTAGGG) region that protect from chromosomal deterioration [4]. Telomerase has been shown to be essential for telomere maintenance and long-term viability [5]. In humans, telomerase activity is limited in the germ line, activated lymphocytes and some types of stem cells [6, 7]. In most cancer cells, telomerase activity is up-regulated to maintain telomere length related to cell immortality [5]. The level of telomerase activity might also be of prognostic relevance [8, 9]. In MDS, the T-cells are characterized by skewed repertoire distribution and form suppressive lymphoid aggregates within the bone marrow of some patients where they can directly suppress hematopoiesis [10, 11].

In humans, the active telomerase complex is composed of at least two components: hTR, which contains the template for reverse transcription, and hTERT, which is the rate limiting catalytic subunit of telomerase [12]. Except for full length hTERT, the hTERT transcript is subjected to numerous alternative splicing events in several tissues and cell lines. To date, more than twenty different alternative splice variants have been reported, including both deletions and insertions [13]. The most extensively studied alternative splice variants involve deletions at two splicing sites: the α splicing site with a deletion of 36 nucleotides in exon 6 and the β site resulting in a 182 nucleotide deletion in exons 7 and 8 [14, 15]. Splicing at the two sites can occur alone or in combination, producing a number of variants with different levels and proportions [16]. Overall, in most studies, α+β- or α+β+ splice variants were most abundant, whereas the α-deletion variants tended to be the lowest isoforms [17, 18]. Alternative splicing of hTERT transcripts represents another regulatory mechanism for telomerase activity. In telomerase-positive immortal cells, it had been shown that the overexpression of α deletion variant had a significant inhibitory effect on enzyme activity [16, 19]. In melanoma, overexpression of the hTERT β deletion variant was also reported to be involved in the negative regulation of telomerase activity [20, 21]. Gaining further insight into hTERT and hTERT alternative splicing in MDS may be important in understanding telomerease regulation during cancer initiation, differentiation and progression.

In the present study, we compare the differences in telomere length, telomerase activity, hTERT and hTERT alternative splice variants in unstimulated T-cells among different MDS subgroups and healthy controls. We also analyzed the correlation between these parameters and their association with the clinicopathological features of MDS patients.

## RESULTS

### Clinical details of MDS patients

As shown in Table 1, twenty MDS patients (mean age 64, range 39-74 years) were enrolled in this study, including 10 females and 10 males. Twenty healthy controls (mean age 61, range 35-75 years) were also included, consisting of 11 females and 9 males. MDS patients and controls were equally matched based on both age and sex (p=0.12 and 0.79, respectively). MDS patients were classified as refractory anemia with or without ringed sideroblast (RA, RARS) (n =4, 20%), refractory cytopenia with multilineage dysplasia (RCMD) (n =9, 45%), or refractory anemia with excess blasts (RAEB) 1/2 (n =7, 35%) based on WHO classification criteria. According to the IPSS, 7 (35%) patients were low risk, 6 (30%) intermediate-1, 5(25%) intermediate-2, and 2 (10%) high risk. Additionally, 12 (60%) MDS patients showed abnormal cytogenetics.

**Table 1:** Clinical characteristics of MDS cases and controls

Characteristics of cases and controls			
Age			
Case:Controls	Mean age (range)		p-value
Controls (n=20)	61 (35-75)		
MDS Cases (n=20)	64 (39-74)		0.12
Sex (Male/Female)	N (M/F)	% (M/F)	
Controls (n=20)	9/11	45/55	
MDS Cases (n=20)	10/10	50/50	0.79
**Clinical characteristics of MDS cases**	n	%	
IPSS* classification			
Low	7	35.0	
Intermediate-1	6	30.0	
Intermediate-2	5	25.0	
High	2	10.0	
Cytogenetics			
Normal	8	40.0	
Abnormal	12	60.0	
WHO^##^ MDS subtype			
RA^#^ with ringed sideroblasts (RARS)	4	20.0	
RCMD^$^	9	45.0	
RA with excess blasts (RAEB)-1^%^	4	20.0	
RAEB-2	3	15.0	

* IPSS=International Prognostic Scoring System;

## WHO=World Health Organization;

# RA=Refractory anemia

$ RCMD=Refractory cytopenia with multilineage dysplasia including patients with (n=1) or without ringed sideroblasts.

% Refractory anemia with excess blasts −1 and 2

### Shorter telomere length in MDS patients

**Figure 1:**
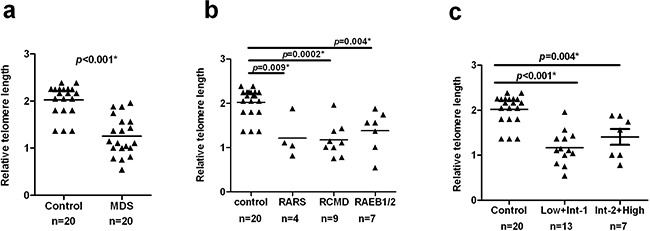
Shorter telomere length in MDS patients CD3^+^ T-cells were purified from PBMCs of MDS patients (n = 20) and healthy controls (n = 20). Telomere length was detected by quantitative PCR with 293 T-cells used as a calibrator. Results were analyzed using the ΔΔCt method. Differences of the telomere length in T-cells were compared using the Wilcoxon rank-sum test. **a.** The MDS cases had a significantly shorter telomere length than healthy controls in T-cells (*p* < 0.001). **b.** Based on WHO classification criteria, MDS patients were separated into three distinct subgroups: RARS, RCMD, RAEB1/2. Each subgroup was found to have shorter telomere length compared with healthy controls (RARS: *p*=0.009; RCMD, *p*=0.0002; RAEB1/2, *p*=0.004, respectively). **c.** According to IPSS, patients were divided into two groups: Low+Int-1 and Int-2+High. Telomere lengths of both groups were shorter than controls (Low+Int-1, *p*<0.001; Int-2+High, *p*=0.004). All tests were considered statistically significant at a significance level of *p* < 0.05.

Results from measurement of relative telomeric DNA in unstimulated T-cells were available for MDS cases and controls. Significantly less telomere DNA was observed in MDS cases (n = 20) (median = 1.127, 95% CI, 1.061–1.446) compared to healthy controls (n = 20) (median =2.165, 95% CI, 1.865–2.175) (*p* <0.001, Figure 1a).

Additional analyses were aimed at further elucidating differences in telomere length of T-cells between different subgroups of MDS cases using both WHO and IPSS classification criteria. Compared to controls, all the subgroups had a significantly shorter telomere length (WHO: RARS, *p*=0.009; RCMD, *p*=0.0002; RAEB1/2, *p*=0.004, respectively, Figure 1b. IPSS: Low+Int-1, *p*<0.001; Int-2+High, *p*=0.004, Figure 1c), but we did not find significant differences among distinct WHO(*p*=0.5760)or IPSS (*p*=0.3830) subtypes of MDS (Table 2).

**Table 2:** Clinical characteristics and telomerase measurements in CD3^+^ T-cells myelodysplastic syndrome (MDS) cases

N=20			TRL			TA			Total hTERT			hTERTα+β+			hTERT α+β-		
Characteristic	n	%	Median	95%CI	P-value	Median	95%CI	P-value	Median	95%CI	P-value	Median	95%CI	P-value	Median	95%CI	P-value
**WHO subtype**																	
RARS	4	20	1.076(0.4764-1.949)			86.79(8.988-142.0)			8.37(0.7154-13.80)			2.881(0.8582-4.817)			12.49(−2.521-34.64)		
RCMD	9	45	1.108(0.8858-1.459)			74.5(56.06-86.40)			3.700(2.229-6.900)			2.385(0.9473-4.483)			6.419(4.048-11.45)		
RAEB1/2	7	35	1.560(0.9522-1.807)		0.576	53.5(32.69-94.38)		0.7631	5.700(3.531-8.115)		0.2986	1.647(0.2668-3.723)		0.4288	7.333(5.520-14.09)		0.1807
**IPSS score**																	
Low+Int-1	13	65	1.108(0.9446-0.9787)			73.5(59.67-87.91)			5.634(4.084-7.880)			2.017(1.467-2.816)			8.489(5.939-15.64)		
Int-2+High	7	35	1.560(1.395-1.836)		0.383	74.5(27.08-95.35)		0.4278	5.287(1.709-7.581)		0.4281	2.012(0.4298-5.831)		0.9053	5.868(3.265-12.89)		0.5003

95%CI=95%confidence interval;

TA=telomerase activity;

RTL=relative telomere length;

hTERT=human telomerase reverse transcriptase;

WHO = World Health Organization;

IPSS = International Prognostic Scoring System.

### Increased telomerase activity and overexpression of hTERT mRNA in MDS patients

**Figure 2:**
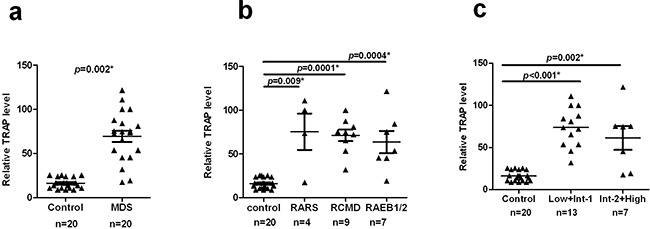
Increased telomerase activity in MDS patients CD3+ T-cells were obtained as described above. Telomerase activity was measured using TRAP assay. **a.** Telomerase activity is significantly increased in MDS versus controls (*p* = 0.002). **b** and **c.** Compared with the control group, different MDS subgroups showed significantly higher telomerase activity (WHO: RARS, *p*=0.009; RCMD, *p*=0.0001; RAEB1/2, *p*=0.0004, respectively. IPSS: Low+Int-1, *p*<0.001; Int-2+High, *p*=0.001). All tests were considered statistically significant at a significance level of *p* < 0.05.

Telomerase function in unactivated T-cells in MDS was then examined using the TRAP assays, as described previously for activated T-cells. Comparing the T-cells from MDS cases and controls, we observed a significant increase in telomerase activity in cases as shown in Figure 2a (median=74.06, 95% CI, 56.04–82.73 in cases and median =15.11, 95% CI, 13.30–19.07 in controls) (*p* = 0.002). Further analysis showed no significant differences in T-cell telomerase activity among different patient subgroups (WHO: *p* = 0.7631;IPSS: *p* = 0.4278, Table 2) and significantly higher enzymatic activity in all subgroups compared to controls (WHO: RARS, *p*=0.009; RCMD, *p*=0.0001; RAEB1/2, *p*=0.0004, respectively, Figure 2b. IPSS: Low+Int-1, *p*<0.001; Int-2+High, *p*=0.001, Figure 2c).

**Figure 3:**
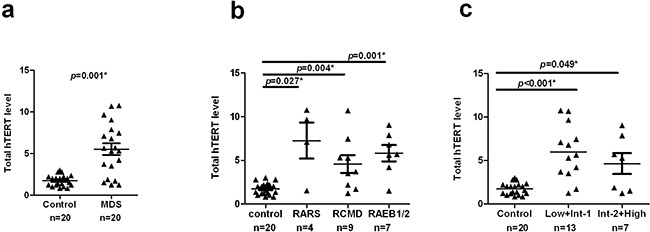
Increased hTERT mRNA expression levels in MDS patients hTERT mRNA expression was quantified by qRT-PCR normalized to 18s ribosomal RNA. **a.** The amount of total hTERT level was compared between MDS cases and controls. MDS cases showed a significantly higher hTERT levels relative to controls (*p*=0.001). **b.** Similar results were observed between each MDS subgroup and controls (RARS: *p*=0.027; RCMD, *p*=0.004; RAEB1/2, *p*=0.001, respectively). **c.** The two IPSS subgroups also showed significantly higher hTERT levels than controls (Low+Int-1, *p*<0.001; Int-2+High, *p*=0.049). All tests were considered statistically significant at a significance level of *p* < 0.05.

hTERT mRNA expression closely correlates with enzymatic activity of telomerase. Our results show that the amount of hTERT mRNA is significantly higher in MDS cases (median =5.468, 95% CI, 4.046–6.982) compared with healthy controls (median =1.784, 95% CI, 1.431–2.068) (*p* = 0.001, Figure 3a). Moreover, distinct MDS subgroups showed significantly higher hTERT expression (WHO: RARS, *p*=0.027; RCMD, *p*=0.004; RAEB, *p*=0.001, respectively, Figure 3b. IPSS: Low+Int-1, *p*<0.001; Int-2+High, *p*=0.049, Figure 3c).

### Increased expression of hTERT mRNA splice variants in MDS patients

**Figure 4:**
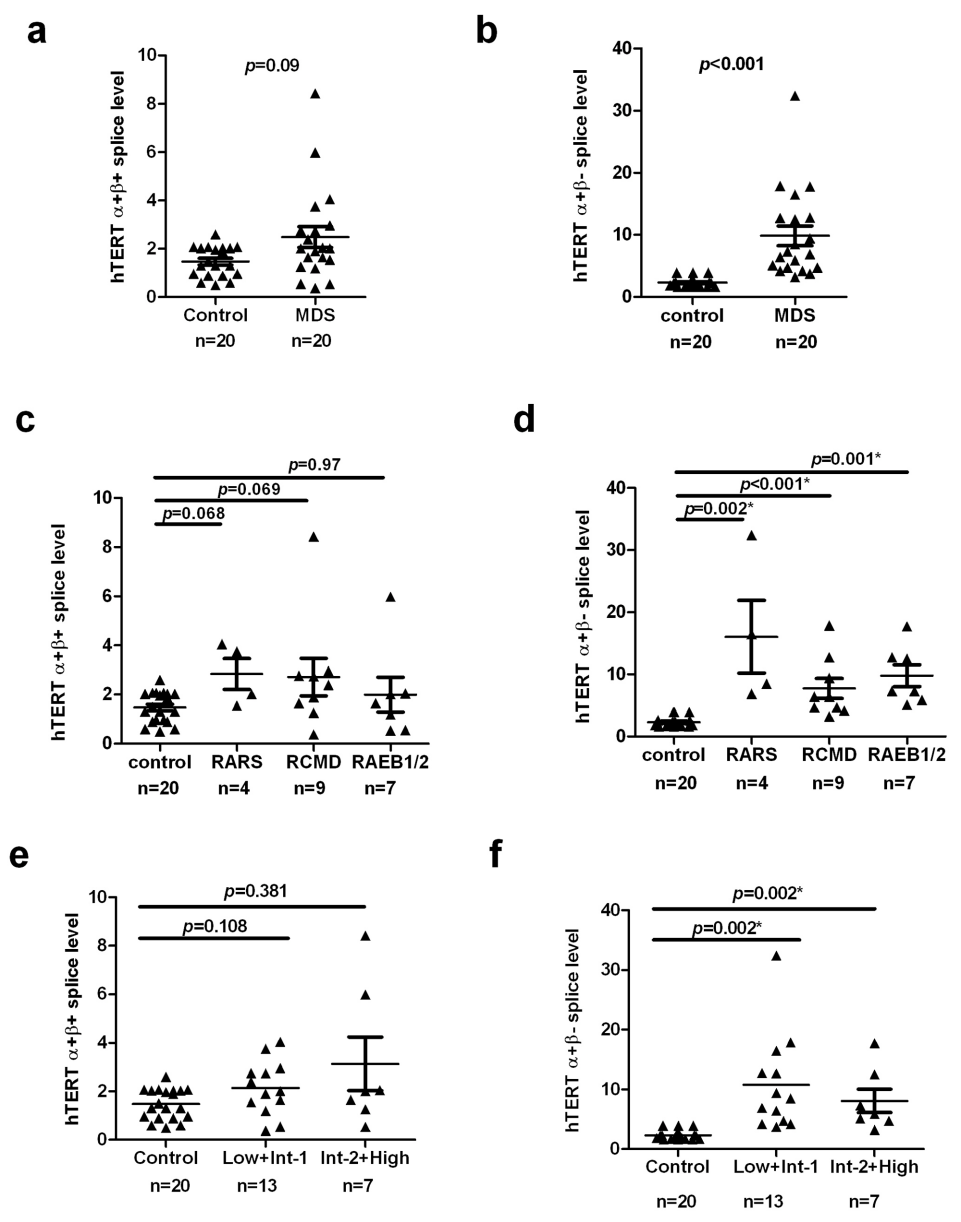
hTERT splice variants analysis in MDS patients and controls Case–control differences for hTERT splice variants in T-cells were compared using the Wilcoxon sum rank test. **a.** The difference of hTERT α+β+ splice variant was not significant between MDS cases and controls (*p*=0.09). **b.** hTERT α+β- splice variant of T-cells was significantly higher in MDS patients compared to control (*p*<0.001). **c** and **e.** The levels of α+β+ splices variant between each subgroup of MDS patients and controls had no significant difference. **d** and **f.** α+β- splices variant were significantly higher in MDS subgroups than healthy controls (WHO: RARS, *p*=0.002; RCMD, *p* <0.001; RAEB1/2, *p*=0.001, respectively. IPSS: Low+Int-1, *p*=0.002; Int-2+High, *p*=0.002). *p* < 0.05 was considered statistically significant.

hTERT is subjected to numerous alternative splicing events, several of which were reported to correlate with the regulation of telomerase activity [22]. To gain further insight into the expression of hTERT mRNA splice variants in MDS cases, we detected the mRNA levels of the full length hTERT (α+β+) and three additional splice variants (α+β−, α−β+ and α−β−). Our data shows that the α+β+ hTERT splice variant tended to be higher in MDS cases (n=20) (median =2.014, 95% CI, 1.586–3.389) than controls (n=20) (median =1.401, 95% CI, 1.185–1.769), but this difference was not statistically significant (*p*=0.09; Figure 4a). In contrast, the α+β− splice variant was expressed at significantly higher levels in cases than controls (MDS, n=20, median = 7.101, 95% CI, 6.497–13.18 vs control, n=20, median = 2.204, 95% CI, 1.947–2.668) (*p*<0.001; Figure 4b).

When we performed analysis of hTERT splice variant levels between different subgroups and healthy controls, each subgroup was found to display significantly higher amount of the α+β− splice variant (WHO: RARS, *p*=0.002; RCMD, *p*<0.001; RAEB1/2, *p*=0.001, respectively, Figure 4d; IPSS: Low+Int-1, *p*=0.002; Int-2+High, *p*=0.002, Figure 4f). Although these subgroups had a higher α+β+ splice level, there were no statically significant differences between cases stratified by WHO and IPSS subtype and controls (WHO: RARS, *p*=0.068; RCMD, *p*=0.069; RAEB1/2, *p*=0.97, respectively, Figure 4c; IPSS: Low+Int-1, *p*=0.108; Int-2+High, *p*=0.381, Figure 4e). Still, no significant differences in α+β+ or α+β− splice levels were observed in multiple comparisons among either WHO (*p* = 0.4288 and 0.1807, respectively) or IPSS (*p* = 0.9053 and 0.5003, respectively) subgroups (Table 2).

### Correlation and clinical relevance of telomerase abnormalities in MDS

We conducted analyses to discover whether there is a relationship between telomere length, telomerase activity, levels of hTERT mRNA and hTERT splice variants (Table 3). Telomere length was not correlated with telomerase activity, total hTERT mRNA level, or levels of α+β+ and α+β− splice variants. However, the amount of telomerase activity is significantly correlated to total hTERT expression (*p*=0.007, r=0.58), but had no obvious relationship with α+β+ or α+β− hTERT splice variants. Expression of the splice variants did not correlate to the total hTERT expression and there was no significant correlation between α+β+ and α+β− hTERT splice variants. In addition, we failed to find any association of telomere length, telomerase activity, total hTERT expression, and levels of hTERT splice variants with clinicopathological parameters of MDS patients (IPSS score, cytogenetics, or WHO subtype) (data not shown).

**Table 3:** Correlation analysis in MDS patients

	TA		Total hTERT		hTERT α+β+		hTERT α+β-	
	r	P value	r	P value	r	P value	r	P value
RTL	−0.463	0.06	−0.198	0.445	0.117	0.653	−0.050	0.844
TA	—	—	0.58	0.007	−0.130	0.619	0.284	0.26

The relationships between RTL/TA and levels of hTERT or hTERT splice variants were further analyzed. Correlations were assessed using the Spearman correlation coefficient. *P* values <0.05 were considered statistically significant.

TA=telomerase activity;

RTL=relative telomere length;

hTERT=human telomerase reverse transcriptase.

## DISCUSSION

In our previous study, we reported the role of hTERT and its splice variants in regulating telomerase activity of PBMCs in MDS patients [23]. We also previously reported a deficiency in total inducible hTERT mRNA expression and hTERT activity in T-cells after stimulation of the T-cell receptor (TCR) [24] that correlated with a loss of proliferative function. Basal levels of hTERT and hTERT splice variants in T-cells of MDS cases have not been investigated. Previous studies reported telomerase induction in bone marrow and mononuclear cells from peripheral blood of MDS patients [25, 26]. In the present study using unactivated T-cells purified from peripheral blood, we found that hTERT mRNA and telomerase activity were significantly increased in MDS cases compared to age and sex-matched healthy donors. Total hTERT mRNA expression is the rate-limiting step of telomerase activity [27–29]. In acute myelogenous leukemia (AML) cells, high telomerase activity correlated well with hTERT expression [30]. These observations, along with our previous data in activated T-cells and PBMCs of MDS [23, 24], suggests that abnormal hTERT transcriptional regulation might serve as the primary mechanism for telomerase dysfunction in both basal and stimulated T-cells.

Splice variants of hTERT have been proposed to represent both diagnostic and prognostic biomarkers in cancer patients [31]. Several studies have correlated hTERT alternative splicing patterns in tumors with histopathological and clinical parameters and with hTERT enzymatic activity. Full length hTERT (α+β+ splicing) has been shown to be strongly associated with tumor development and progression. In many malignant tissues, tumors with disease progression and shorter survival tended to express more of this full-length hTERT transcript. Alternatively benign tumors with better prognosis tended to have less full length hTERT mRNA [18, 32–35]. In PBMCs from patients with chronic myelogenous leukemia (CML), levels of both full-length and deletion hTERT transcripts (α+β-) were higher in progressive than in non-progressive patients and the levels of full-length form increased with clinical stage [36]. In Non-Small Cell Lung Cancer (NSCLC) tumors, the expression of α+β+ variants correlated with overall survival and disease-free survival and may be an independent negative prognostic factor in these patients [18]. Although we did not find any correlation of telomere, telomerase, hTERT mRNA or its splice levels with histopathological and clinical features of MDS, the significant increase in hTERT activity and hTERT mRNA in unstimulated T-cells in patients compared to controls provides supportive evidence for possible role for telomere deregulation in MDS pathobiology.

To determine if altered splicing may be responsible for differential hTERT expression and activity, we examined various hTERT splice variants known to impact hTERT function [16, 18, 32]. Interestingly, hTERT α+β+ transcript was not significantly different between MDS cases and controls; whereas, the α+β- hTERT mRNA splice variant was significantly higher. Splice variants of hTERT may have a regulatory role in telomerase activity. In melanoma and breast cancer cells, the hTERT α+β- variant was reported to be involved in negative regulation of telomerase activity [20, 21]. In the majority of human immortal and cancer cell lines, α+β+ mRNA expression correlates with telomerase activity [17]. Since there was an accumulation of the α+β- variant in MDS T-cells, this may contribute to the reduction in enzymatic activity. However, in our current study, we did not find an obvious relationship between hTERT α+β+ or α+β- splice variants and telomerase activity. A putative explanation for these findings may be that the regulation of telomerase activity by hTERT splice variants is tumor-specific and that there could be additional splice variants present in T-cells of MDS patients that were not investigated. Different types of tumors have different profiles of hTERT transcript expression and telomerase activity regulators [37]. Except for α+β+ and α+β- splice variants, many studies also suggest that hTERT α deletion variant have a dominant negative effect on telomerase activity in telomerase-positive immortal cells [16, 19]. Interestingly, we failed to detect both α-β+ and α-β- splice variants in T-cells in this study suggesting that tissue-specific hTERT regulation occurs. Additionally, evidence suggested that transcriptional regulation of hTERT was not sufficient to account for telomerase activity in human lymphocytes, indicating a likely role of posttranscriptional factors in the control of enzyme function [38].

Telomere length shortening has been routinely observed in many studies of MDS patients [33, 36], and our results confirm this disease feature in unstimulated T-cells. In studies like ours where telomere length and telomerase activity have been concurrently studied, there has been no correlation suggesting that the increase in hTERT is insufficient to perform telomere repair and stabilize telomere loss in this disease [6]. It is possible that the higher basal level of hTERT mRNA may reflect activity at the stem cell level where telomere repair is needed to maintain chromosomal stability in highly proliferative cells.

Telomerase can compensate for telomere shortening and telomerase activity is expressed at a low level in normal peripheral leukocytes [39]. Telomerase activity varies in different lymphocyte subsets [40]. The changes of telomerase activity did not parallel within different T-cell subsets when exposed to cortisol [41]. Whether the telomere shortening in naïve T cells instead of memory T cell is due to immune cell type-specific telomerase activity is unknown, and it seems of interest to clarify this issue in further research.

In summary, our study compared basal levels of telomere length, telomerase activity, total hTERT mRNA and hTERT alternative splice variants in unstimulated T-cells between MDS patients and controls. The results indicate that both overall hTERT and α+β− splice variant are promising biomarkers for MDS, and total hTERT mRNA may account for telomerase activity in peripheral T-cells. But replication in more populations and mechanism investigation are necessary in future studies. The function and biological role of hTERT in basal and in activated T-cells also remain to be clarified in the further studies.

## MATERIALS AND METHODS

### Patients and healthy controls

After obtaining informed consent, twenty histologically confirmed MDS patients participated in a study to obtain peripheral blood samples at the Tianjin Medical University Cancer Institute and Hospital, Tianjin, China. These patients were classified into different subgroups according to the World Health Organization (WHO) and International Prognostic Scoring System (IPSS) criteria. Twenty age and sex-matched healthy donors participated as controls and peripheral blood was obtained from each of these individuals. Characteristics of cases and controls are summarized in Table 1.

### Isolation of CD3^+^ T-cells

Peripheral blood mononuclear cells (PBMCs) were isolated with Ficoll-Hypaque (Chinese Academy of Medical Sciences, China) gradient centrifugation from both MDS patients and healthy controls followed by purification of CD3^+^ T-cells using positive selection by CD3 microbeads (Miltenyi Biotec). The purity of the CD3^+^ T-cells was evaluated by flow cytometry and all samples were confirmed to be above 97% CD3^+^.

### Telomeric DNA measurements

Relative telomere length was measured by the amount of telomeric DNA present in purified T-cells and calculated as described previously [24]. Briefly, DNA was extracted from CD3^+^ T-cells with the Pure Link Genomic DNA Kits (Tiangen, Beijing, China). Hemoglobin was used as internal control. SYBR Green PCR Master Mix (Applied Biosystems) for telomere and hemoglobin were added into a 96-well format. The primer sequences for telomere amplification were Tel-1: 5′CGG TTT GTT TGG GTT TGG GTT TGG GTT TGG GTT TGG GTT-3′; Tel-2: 5′GGC TTG CCT TAC CCT TAC CCT TAC CCT TAC CCT TAC CCT-3′; hgb1: 5′GCTTCTGACACAACTGTGTTCACT AGC-3′ and hgb2: 5′CACCAA CTTCATCCACGTTCACC-3′. A five-point standard curve was obtained using genomic DNA ranged from 0.08 ng to 50 ng of 293T cells in each 96-well plate. The relative telomere length was calculated as a ratio of the telomere repeat copy number (T) to the single-gene copy number (S) using an Applied Biosystems 7500 HT PCR system. For each sample, the relative T/S ratio (−ΔCt) was determined by subtracting the T/S ratio of the 10.0 ng standard curve point from the T/S ratio of each unknown sample. Experiments were repeated in triplicate.

### Telomeric repeat amplification protocol (TRAP) assay

Telomerase activity was measured based on the telomeric repeat amplification protocol (TRAP). Briefly, a 20 μl reaction system includes SYBR Green PCR Master Mix (Applied Biosystems), cell extracts and primer for telomerase TS and anchored return primer ACX. Primers sequences were as follows: TS: AATCCGTCGAGCAGAGTT; ACX: GCGCGGCTTACCCTTACCCTTACCCTA A CC. The PCR was performed in a 96-well plate on an ABI PRISM 7500 Thermal cycler (Applied Biosystems). Standard curves for each assay were obtained with 293T cell extracts. Inactivated samples and lysis buffer were assayed on a 96 well plate in triplicate. The percentage of telomerase activity was compared between samples and 293T cells.

### Detection of hTERT mRNA expression

Total cellular RNA was isolated from T-cells with TRIzol (Invitrogen, Carlsbad, CA, USA). RNA was reversed-transcribed using cDNA reverse transcription kit (TakaRa, Japan) according to the manufacturer's instructions. qRT-PCR amplification was performed in a 7500 real-time PCR amplifier (Applied Biosystems, USA) using 1× Taqman mixture, 1× primer of hTERT or 18S rRNA(Applied Biosystems, USA). Expression of hTERT was normalized by using 18S as internal control. The relative levels of gene expression were analyzed using ΔΔCt method. Each qRT-PCR reaction was repeated in triplicate for stable results.

### Detection of hTERT splice variants

Total RNA was extracted from CD3^+^ T-cells, as described above, and reversed transcribed to cDNA with cDNA reverse transcription kit (TaKaRa, Japan). Primers sequences for corresponding splice variants and TaqMan probes used in the present study are shown in Table 4. Relative hTERT splice variants gene expression was measured by a qRT-PCR performed in a 7500 real-time PCR amplifier (Applied Biosystems, USA) with 18S rRNA used as internal control. The cycling protocol for all splice variants consisted of a 5-min initial denaturation at 95°C and a 50 cycle amplification (95°C for 10 s, annealing temperature at 65°C for 20 s, and extension at 72°C for 20 s). The expression level of hTERT for each sample was calculated with the ΔΔCt method.

**Table 4:** Sequences of primers and TaqMan probe used in the present study

Use	Name	Sequence^a^ (5′-3′)	T_m,_(°C)
Forward primer	TE 1/2	TCA AGG TGG ATG TGA CGG G	59.2
Forward primer	TER3	CCT GAG CTG TAC TTT GTC AAG GA	59.0
Reverse primer	KAT4b	GGA CTT GCC CCT GAT GCG	61.7
Reverse primer	TER2	GGC ACT GGA CGT AGG ACG TG	61.0
Taqman probe	Probe	6FAM - CGT GTT CTG GGG TTT GAT GAT GCT GGC GA - TMR	74.5

### Statistical analysis

Using unactivated T-cells purified from peripheral blood, descriptive statistics including median and 95% confidence interval (CI) were calculated for continuous variables including telomere length, telomerase activity, hTERT mRNA expression and expression of hTERT splice variants. Comparisons between these continuous variables between cases and controls were performed by the Wilcoxon rank-sum test. Patients were classified by WHO criteria and by lower-risk (low/intermediate-1) or higher-risk (intermediate 2/high) based on their IPSS score, and telomere variables were compared across these clinical characteristics. Spearman's correlation coefficients (r) were calculated to assess associations between telomere length, telomerase activity, total hTERT and hTERT splice variant expression. All tests were two-sided and statistical analyses were performed with SPSS 19.0 software package (SPSS Inc., Chicago, IL, USA). *p* value <0.05 was considered statistically significant.

## References

[1] Yang L, Qian Y, Eksioglu E, Epling-Burnette PK, Wei S (2015). The inflammatory microenvironment in MDS. Cell Mol Life Sci.

[2] Ohyashiki JH, Ohyashiki K, Fujimura T, Kawakubo K, Shimamoto T, Iwabuchi A, Toyama K (1994). Telomere shortening associated with disease evolution patterns in myelodysplastic syndromes. Cancer Res.

[3] Ohyashiki JH, Iwama H, Yahata N, Ando K, Hayashi S, Shay JW, Ohyashiki K (1999). Telomere stability is frequently impaired in high-risk groups of patients with myelodysplastic syndromes. Clin Cancer Res.

[4] Young NS (2010). Telomere biology and telomere diseases: implications for practice and research. Hematology / the Education Program of the American Society of Hematology American Society of Hematology Education Program.

[5] Wang C, Yu J, Yuan K, Lan J, Jin C, Huang H (2010). Plk1-mediated mitotic phosphorylation of PinX1 regulates its stability. Eur J Cell Biol.

[6] Broccoli D, Young JW, de Lange T (1995). Telomerase activity in normal and malignant hematopoietic cells. Proc Natl Acad Sci U S A.

[7] Wright WE, Piatyszek MA, Rainey WE, Byrd W, Shay JW (1996). Telomerase activity in human germline and embryonic tissues and cells. Dev Genet.

[8] Keller G, Brassat U, Braig M, Heim D, Wege H, Brummendorf TH (2009). Telomeres and telomerase in chronic myeloid leukaemia: impact for pathogenesis, disease progression and targeted therapy. Hematol Oncol.

[9] Sommerfeld HJ, Meeker AK, Piatyszek MA, Bova GS, Shay JW, Coffey DS (1996). Telomerase activity: a prevalent marker of malignant human prostate tissue. Cancer Res.

[10] Sloand EM, Melenhorst JJ, Tucker ZC, Pfannes L, Brenchley JM, Yong A, Visconte V, Wu C, Gostick E, Scheinberg P, Olnes MJ, Douek DC, Price DA, Barrett AJ, Young NS (2011). T-cell immune responses to Wilms tumor 1 protein in myelodysplasia responsive to immunosuppressive therapy. Blood.

[11] Sloand EM, Kim S, Fuhrer M, Risitano AM, Nakamura R, Maciejewski JP, Barrett AJ, Young NS (2002). Fas-mediated apoptosis is important in regulating cell replication and death in trisomy 8 hematopoietic cells but not in cells with other cytogenetic abnormalities. Blood.

[12] Nakayama J, Ishikawa F (1998). Structure and regulation mechanisms of telomerase. [Article in Japanese]. Nihon Rinsho.

[13] Bollmann FM (2013). Physiological and pathological significance of human telomerase reverse transcriptase splice variants. Biochimie.

[14] Kilian A, Bowtell DD, Abud HE, Hime GR, Venter DJ, Keese PK, Duncan EL, Reddel RR, Jefferson RA (1997). Isolation of a candidate human telomerase catalytic subunit gene, which reveals complex splicing patterns in different cell types. Hum Mol Genet.

[15] Wick M, Zubov D, Hagen G (1999). Genomic organization and promoter characterization of the gene encoding the human telomerase reverse transcriptase (hTERT). Gene.

[16] Yi X, White DM, Aisner DL, Baur JA, Wright WE, Shay JW (2000). An alternate splicing variant of the human telomerase catalytic subunit inhibits telomerase activity. Neoplasia.

[17] Yi X, Shay JW, Wright WE (2001). Quantitation of telomerase components and hTERT mRNA splicing patterns in immortal human cells. Nucleic Acids Res.

[18] Mavrogiannou E, Strati A, Stathopoulou A, Tsaroucha EG, Kaklamanis L, Lianidou ES (2007). Real-time RT-PCR quantification of human telomerase reverse transcriptase splice variants in tumor cell lines and non-small cell lung cancer. Clin Chem.

[19] Colgin LM, Wilkinson C, Englezou A, Kilian A, Robinson MO, Reddel RR (2000). The hTERTalpha splice variant is a dominant negative inhibitor of telomerase activity. Neoplasia.

[20] Lincz LF, Mudge LM, Scorgie FE, Sakoff JA, Hamilton CS, Seldon M (2008). Quantification of hTERT splice variants in melanoma by SYBR green real-time polymerase chain reaction indicates a negative regulatory role for the beta deletion variant. Neoplasia.

[21] Listerman I, Sun J, Gazzaniga FS, Lukas JL, Blackburn EH (2013). The major reverse transcriptase-incompetent splice variant of the human telomerase protein inhibits telomerase activity but protects from apoptosis. Cancer Res.

[22] Ulaner GA, Hu JF, Vu TH, Giudice LC, Hoffman AR (1998). Telomerase activity in human development is regulated by human telomerase reverse transcriptase (hTERT) transcription and by alternate splicing of hTERT transcripts. Cancer Res.

[23] Dong W, Qian Y, Yang L (2014). Telomerase, hTERT and splice variants in patients with myelodysplastic syndromes. Leuk Res.

[24] Yang L, Mailloux A, Rollison DE, Painter JS, Maciejewski J, Paquette RL, Loughran TP, McGraw K, Makishima H, Radhakrishnan R, Wei S, Ren X, Komrokji R, List AF, Epling-Burnette PK (2013). Naive T-cells in myelodysplastic syndrome display intrinsic human telomerase reverse transcriptase (hTERT) deficiency. Leukemia.

[25] Gurkan E, Tanriverdi K, Baslamisli F (2005). Telomerase activity in myelodysplastic syndromes. Leuk Res.

[26] Briatore F, Barrera G, Pizzimenti S, Toaldo C, Casa CD, Laurora S, Pettazzoni P, Dianzani MU, Ferrero D (2009). Increase of telomerase activity and hTERT expression in myelodysplastic syndromes. Cancer Biol Ther.

[27] Xu D, Gruber A, Bjorkholm M, Peterson C, Pisa P (1999). Suppression of telomerase reverse transcriptase (hTERT) expression in differentiated HL-60 cells: regulatory mechanisms. Br J Cancer.

[28] Tominaga T, Kashimura H, Suzuki K, Nakahara A, Tanaka N, Noguchi M, Itabashi M, Ohkawa J (2002). Telomerase activity and expression of human telomerase catalytic subunit gene in esophageal tissues. J Gastroenterol.

[29] Kirkpatrick KL, Clark G, Ghilchick M, Newbold RF, Mokbel K (2003). hTERT mRNA expression correlates with telomerase activity in human breast cancer. Eur J Surg Oncol.

[30] Xu D, Gruber A, Peterson C, Pisa P (1998). Telomerase activity and the expression of telomerase components in acute myelogenous leukaemia. Br J Haematol.

[31] Brinkman BM (2004). Splice variants as cancer biomarkers. Clin Biochem.

[32] Wang Y, Kowalski J, Tsai HL, Marik R, Prasad N, Somervell H, Lo PK, Sangenario LE, Dyrskjot L, Orntoft TF, Westra WH, Meeker AK, Eshleman JR, Umbricht CB, Zeiger MA (2008). Differentiating alternative splice variant patterns of human telomerase reverse transcriptase in thyroid neoplasms. Thyroid.

[33] Wang Y, Meeker AK, Kowalski J, Tsai HL, Somervell H, Heaphy C, Sangenario LE, Prasad N, Westra WH, Zeiger MA, Umbricht CB (2011). Telomere length is related to alternative splice patterns of telomerase in thyroid tumors. Am J Pathol.

[34] Hartmann U, Brummendorf TH, Balabanov S, Thiede C, Illme T, Schaich M (2005). Telomere length and hTERT expression in patients with acute myeloid leukemia correlates with chromosomal abnormalities. Haematologica.

[35] Fan Y, Liu Z, Fang X, Ge Z, Ge N, Jia Y, Sun P, Lou F, Bjorkholm M, Gruber A, Ekman P, Xu D (2005). Differential expression of full-length telomerase reverse transcriptase mRNA and telomerase activity between normal and malignant renal tissues. Clin Cancer Res.

[36] Palma M, Parker A, Hojjat-Farsangi M, Forster J, Kokhaei P, Hansson L, Osterborg A, Mellstedt H (2013). Telomere length and expression of human telomerase reverse transcriptase splice variants in chronic lymphocytic leukemia. Exp Hematol.

[37] Petrenko AA, Korolenkova LI, Skvortsov DA, Fedorova MD, Skoblov MU, Baranova AV, Zvereva ME, Rubtsova MP, Kisseljov FL (2010). Cervical intraepithelial neoplasia: Telomerase activity and splice pattern of hTERT mRNA. Biochimie.

[38] Liu K, Schoonmaker MM, Levine BL, June CH, Hodes RJ, Weng NP (1999). Constitutive and regulated expression of telomerase reverse transcriptase (hTERT) in human lymphocytes. Proc Natl Acad Sci U S A.

[39] Weng NP, Hathcock KS, Hodes RJ (1998). Regulation of telomere length and telomerase in T and B cells: a mechanism for maintaining replicative potential. Immunity.

[40] Ouyang Q, Baerlocher G, Vulto I, Lansdorp PM (2007). Telomere length in human natural killer cell subsets. Annals of the New York Academy of Sciences.

[41] Choi J, Fauce SR, Effros RB (2008). Reduced telomerase activity in human T lymphocytes exposed to cortisol. Brain, behavior, and immunity.

